# A Stress Reduction Intervention for Lactating Mothers Alters Maternal Gut, Breast Milk, and Infant Gut Microbiomes: Data from a Randomized Controlled Trial

**DOI:** 10.3390/nu16071074

**Published:** 2024-04-06

**Authors:** Jinyue Yu, Yan Zhang, Jonathan C. K. Wells, Zhuang Wei, Mona Bajaj-Elliott, Dennis Sandris Nielsen, Mary S. Fewtrell

**Affiliations:** 1 Childhood Nutrition Research Group, Population, Policy & Practice Department, UCL Great Ormond Street Institute of Child Health, London WC1N 1EH, UK; jin.yu.16@ucl.ac.uk (J.Y.); jonathan.wells@ucl.ac.uk (J.C.K.W.); 2 Microbiota Division, Department of Gastroenterology and Hepatology, The First Medical Center, Chinese PLA General Hospital, Beijing 100853, China; bellazy1125@gmail.com; 3 Department of Child Healthcare, Beijing Children’s Hospital, Capital Medical University, National Center for Children’s Health, Beijing 100045, China; zhuang.wei.office@aliyun.com; 4 Infection, Immunity & Inflammation Department, UCL Great Ormond Street Institute of Child Health, London WC1N 1EH, UK; m.bajaj-elliott@ucl.ac.uk; 5 Department of Food Science, University of Copenhagen, 1958 Frederiksberg C, Denmark; dn@food.ku.dk

**Keywords:** breastfeeding, gut microbiome, maternal stress, infant weight, mother–infant signaling

## Abstract

Background: This secondary analysis of data from a randomized controlled trial (RCT) investigated how the maternal gut, breast milk, and infant gut microbiomes may contribute to the effects of a relaxation intervention, which reduced maternal stress and promoted infant weight gain. Methods: An RCT was undertaken in healthy Chinese primiparous mother–infant pairs (34^0/7^–37^6/7^gestation weeks). Mothers were randomly allocated to either the intervention group (IG, listening to relaxation meditation) or the control group (CG). Outcomes were the differences in microbiome composition and the diversity in the maternal gut, breast milk, and infant gut at 1 (baseline) and 8 weeks (post-intervention) between IG and CG, assessed using 16S rRNA gene amplicon sequencing of fecal and breastmilk samples. Results: In total, 38 mother–infant pairs were included in this analysis (IG = 19, CG = 19). The overall microbiome community structure in the maternal gut was significantly different between the IG and CG at 1 week, with the difference being more significant at 8 weeks (Bray–Curtis distance R^2^ = 0.04 vs. R^2^ = 0.13). Post-intervention, a significantly lower α-diversity was observed in IG breast milk (observed features: CG = 295 vs. IG = 255, *p* = 0.032); the Bifidobacterium genera presented a higher relative abundance. A significantly higher α-diversity was observed in IG infant gut (observed features: CG = 73 vs. IG = 113, *p* < 0.001). Conclusions: The findings were consistent with the hypothesis that the microbiome might mediate observed relaxation intervention effects via gut–brain axis and entero-mammary pathways; but confirmation is required.

## 1. Introduction

Stress has an influence on the structure of the microbiota community in the gastrointestinal (GI) tract [1,2], potentially through the ‘gut–brain axis’ [3,4]. During lactation, the GI tract microbiome of mothers affected by stress could influence the milk microbiome via entero-mammary trafficking, which refers to the movement of microbiota from the maternal gut to the mammary gland [5,6,7]. Moreover, human breast milk contains bacteria including *Lactic acid bacteria* and *Bifidobacteria* [8,9,10] and several studies have suggested that breast milk microbes influence the infant gut microbiome [11,12,13]. An emerging paradigm indicates that maternal psychological status could be associated with alterations in infant gut microbiome diversity [14] (Figure S1, Supplemental File).

Numerous microbes rapidly colonize the gut of the newborn infant. The early life microbiome is known to influence infant development and gut microbiome imbalances have been linked with an increased risk of certain autoimmune diseases [15,16,17,18,19]. The development of the neonatal gut microbiome can be influenced by multiple factors, including delivery mode (hence differential exposure to the maternal microbiome), feeding method, antibiotic intake in early life, stress, and genetic factors [20,21]. Furthermore, the microbe and human milk oligosaccharide (HMO) content of human breast milk have a profound influence on the early gut colonization of the infant [21,22,23].

Compared to healthy term infants, late preterm infants (gestational age 34^0/7^–36^6/7^ weeks) and infants who are born early term (37^0/7^–37^6/7^ weeks) are a vulnerable group who experience greater challenges when establishing breastfeeding. Moreover, compared to very preterm infants, those delivered between 34^0/7^ and 36^6/7^ weeks are generally physiologically able to establish direct breastfeeding but their mothers often receive sub-optimal lactation support, since the infants may be regarded as generally healthy. This can increase maternal stress, which, in turn, has detrimental effects on breastfeeding. We have shown, in three randomized controlled trials (RCTs) (one in term infants, two in late preterm and early term infants), that a simple relaxation intervention significantly reduces psychosocial stress in breastfeeding women, whilst improving infant weight gain [24,25,26]. The present study analyzed data from one of these RCTs and investigated the role of the microbiome as a potential mediator of the observed intervention effects on weight gain in late preterm and early term infants (born at 34^+0^ to 37^+6^ weeks of gestation). We used 16S rRNA gene sequencing to analyze the microbiome in the maternal gut (fecal), breast milk, and infant gut (fecal) samples.

## 2. Materials and Methods

This trial was registered at clinicaltrials.gov as NCT03674632. URL (accessed on 11 March 2020): https://register.clinicaltrials.gov/prs/app/action/SelectProtocol?sid=S00089VA&selectaction=Edit&uid=U00045UL&ts=2&cx=v9xz2g. Details of the trial design and the main outcomes have been published [26,27]. All mothers completed the 8-week data collection before the COVID-19 lockdown in Beijing.

### 2.1. Study Design and Participants

Healthy, non-smoking primiparous mothers with a singleton infant born between 34^+0^ and 37^+6^ weeks gestation who planned to exclusively breastfeed (EBF) for at least two months were recruited 3–5 days after birth. Mothers or infants with serious health concerns and those who needed a prolonged hospital stay were excluded from the study. Recruitment was conducted prior to the first home visit. Mothers were contacted by research nurses from 24 community clinics affiliated to Beijing Children’s Hospital in Beijing, China. Data collection involved two home visits around 1 week (baseline, pre-intervention) and 8 weeks after birth. To maintain uniformity across study sites, all research assistants and nurses received training before recruitment began. Standard operating procedures for the study were printed and displayed at each center. To address the impact of delivery mode on the infant microbiota, the present study only analyzed data from a subset of participants who delivered vaginally and provided maternal fecal, breast milk, and infant fecal samples at baseline (1 week) and 8 weeks home visits. Ethical approval for the study was obtained from the Research Ethics Committee of University College London (ID: 12681/002) and the Department of Child Health, Beijing Children’s Hospital (ID: 2018-167).

### 2.2. Study Procedures and Randomization

Written informed consent was obtained after the recruitment (3–5 days after birth). Prior to the 1 week home visit (5–12 days after birth), mother–infant pairs were randomly allocated to either the intervention group (IG) or the control group (CG). IG mothers were asked to listen to a guided imagery meditation audio designed for breastfeeding mothers [28] at least once a day and record their use in a diary book; the audio consisted of three parts, as follows: (a) introduction and instruction of using; (b) breastfeeding support (15 min), and (c) guided relaxation imagery (15 min); it could be obtained by scanning the QR code provided at the 1 week home visit. Both CG and IG mothers received standard postpartum care from local clinics, but CG mothers were not aware of the relaxation intervention. We used the Chinese version of the Perceived Stress Scale [29] for the measurement of maternal stress. Details can be found in the main outcome paper [26].

The randomization sequence was computer generated by an independent researcher and stratified by gestational age (34–35 vs. 36–37 weeks), delivery method (vaginal vs. caesarean), and recruitment location. Assignments were stored in sealed, opaque envelopes at Beijing Children’s Hospital. Participants were not told about the randomization until the end of the study; they were aware that the aim of the study is to investigate factors that could optimize breastfeeding outcomes. Nurses who collected the samples were aware of the groups but remained impartial, with no vested interest in the study’s outcome. The research technicians at Novogene, who performed the 16s rRNA sequencing, were blinded to the randomization status of the subjects.

### 2.3. Outcomes and Measures

Outcomes of the present study were the differences in microbiome composition and diversity in the maternal gut, breast milk, and the infant gut at 1 and 8 weeks between IG and CG, assessed uing 16S rRNA gene amplicon sequencing of fecal and breastmilk samples.

Baseline characteristics of the mother–infant pairs were obtained using demographic questionnaires. Sample collection methods are stated in our protocol and main outcome study, which has been previously published [26,27]. The breast milk and maternal fecal samples were collected by mothers following the instructions we provided. Mothers were asked to clean their areolar skin using water/soap before collecting milk samples, put samples in our pre-provided sterile cryotubes in ice bags and store these in their fridge/freezer. Infant samples were collected by the nurse from infant diapers during home visits. All samples were transferred by a specialist transport company in a dry ice cooler box and were stored at −80 °C in the laboratory of Beijing Children’s Hospital until the end of the study. Samples for inclusion in the microbiome analysis were then transported by the same company in a dry ice cooler box to the laboratory of Novogene Technology Inc. (Beijing, China) where the DNA extraction, library preparation, and the 16S rRNA gene amplicon sequencing were performed, using standard procedures.

### 2.4. DNA Extraction, Sequencing, and Data Processing

Frozen breast milk and stool samples from the mother and the stool sample from the child were determined using 16S ribosomal (rRNA) sequencing on an Illumina NovaSeq platform 6000 and 250 bp paired-end reads were generated. Total genome DNA from samples was extracted using the CTAB method.

The sequencing targeted the V4 variable region and was amplified using universal primers (515F-GTGCCAGCMGCCGCGGTAA, 806R-GGACTACHVGGGTWTCTAAT). PCR products were purified with the Qiagen Gel Extraction Kit (Cat. #28004, Qiagen, Hilden, Germany). All samples reached band A “The total amount of PCR product meets the needs of one or more library constructions, which can be used for subsequent library constructions”.

Library preparation was performed using the TruSeq^®^ DNA PCR-Free Sample Preparation Kit (Illumina, San Diego, CA, USA), following the manufacturer’s recommendations and index codes were added. Library quality was assessed on the Qubit@ 2.0 Fluorometer (Thermo Scientific, Waltham, MA, USA) and Agilent Bioanalyzer 2100 system, Santa Clara, CA, USA.

### 2.5. Bioinformatic Workflow of 16S rRNA Gene Amplicon

The primer was removed from the raw sequence data (median 150,333 reads per specimen) and merged paired-end sequencing via FLASH [30] version 1.2.7 to obtain raw tags. These tags were then compared with the Silva database 7 via UCHIME (version 11) to remove chimeras. [31] Quantitative Insights Into Microbial Ecology (QIIME2) version 2022.2.0 was used to forward process analysis. [32] We use q2-dada2 [33] to process sequences into an exact sequence features table. Features presenting less than a single sample and features with a total frequency of less than 10 across all samples were filtered from the feature table. To assign taxonomy to the sequences, q2-feature-classifier [34,35] was used with a pre-trained Naive Bayes classifier via the SILVA rRNA database (version 138.1). [36] To filter out outliers from the sequencing results, features with a total abundance of less than 10 were removed and features that appeared in only two samples were removed. Of the 228 samples sequenced, 5729 of 22,610 features remained after these standard quality filtering methods for the following microbiota analysis. Moreover, we provide a p-sampling depth of 80,000 to subsample the counts in each sample without replacement, so that each sample in the resulting table has a total count of 80,000.

### 2.6. Statistical Analysis

Statistical analyses were conducted using R (version 4.12), and SPSS (version 26.0). We compared baseline characteristics, maternal stress, and infant weight gain data between the 38 selected mother–infant pairs and the remainder of the 96 mother–infant pairs were not involved in this analysis. We compared the diversity differences in maternal fecal, breast milk, and infant fecal samples between IG and CG at 1 and 8 weeks. The differences in α-diversity between IG and CG were examined using the Wilcoxon rank-sum test using observed features. Differences in β-diversity between IG and CG were examined using Bray–Curtis dissimilarity metrics presented on a principal coordinates analysis plot (PCoA) and with differences between groups being determined using ANOSIM. The relative abundance of the top 15 most abundant genera in all samples were examined and statistical differences between the IG and CG samples were compared using the Wilcoxon rank-sum test and FDR adjusted *p*-value.

To determine the abundance of specific bacteria and potential associations with maternal stress and infant weight gain, Spearman’s rank correlation was used and results were presented in heatmaps. A standard *p*-value of <0.05 was considered statistically significant for all analyses.

## 3. Results

### 3.1. Study Population and Baseline Characteristics

**Figure 1:**
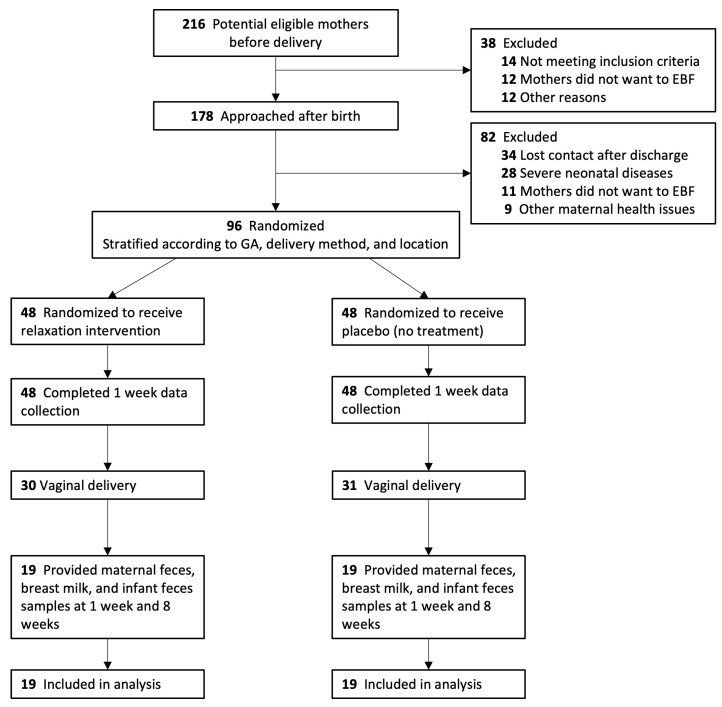
CONSORT flowchart of the randomized controlled trial. Notes: GA = gestational age, EBF = exclusive breastfeeding.

The original trial was conducted from October 2018 to October 2020. Of the 96 mother–infant pairs enrolled in the original trial, 38 eligible pairs were included in this secondary analysis (Figure 1, IG = 19, CG = 19); those 38 eligible pairs were recruited from February 2019 to January 2020. All mothers followed the traditional Chinese postpartum confinement practice during the data collection period. There were no significant differences in baseline characteristics between those who were included in the microbiome analysis and those that were not (Table S1 in Supplemental File). All 228 samples (38 maternal fecal, breast milk, and infant fecal samples at 1 and 8 weeks) were analyzed (rarefaction curves are shown in Figure S2). Distinct bacterial communities were observed in maternal gut, breast milk, and infant gut samples (Figure S3 in Supplemental File). Demographic characteristics of the participants are outlined in Table 1; there were no significant differences in participant characteristics between the IG and CG at baseline.

### 3.2. Microbiome Composition and Diversity in the Maternal Gut

**Figure 2:**
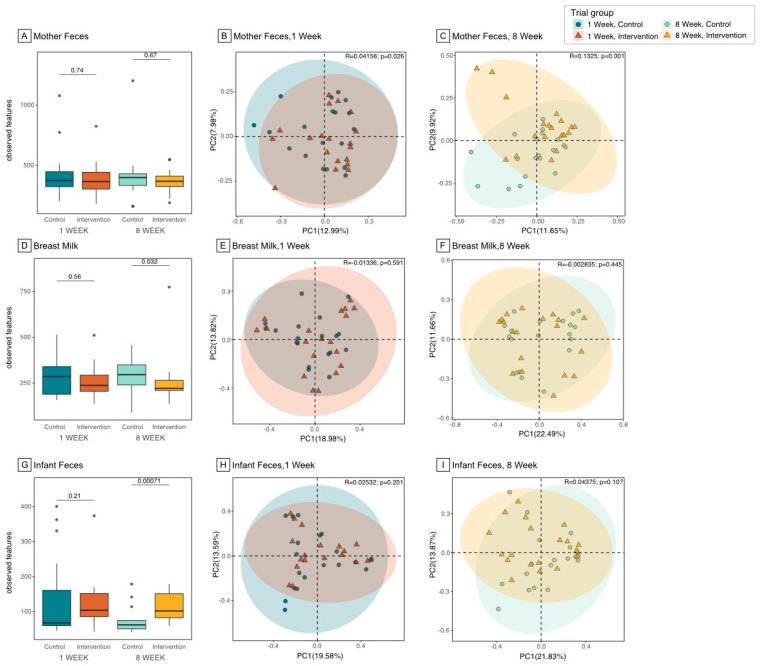
Microbiome diversity in maternal gut, breast milk, and infant gut samples. Notes: Gut microbiome diversity was assessed by analyzing the microbiome composition in fecal samples and breast milk. Differences in maternal gut between intervention and control group at 1 and 8 weeks were shown in (**A**) (α-diversity, within sample diversity), (**B**,**C**) (β-diversity, between sample diversity); differences in breast milk were shown in, (**D**) (α-diversity), (**E**,**F**) (β-diversity); differences in infant gut were shown in (**G**) (α-diversity), (**H**,**I**) (β-diversity). Difference in α−diversity was assessed using the Wilcoxon rank-sum test based on observed features. Differences in β-diversity were presented using a principal coordinates analysis plot (PCoA) based on Bray-Curtis distance matrix.

The number of observed bacterial taxa (observed features) in the maternal gut was not significantly different between IG and CG at both 1 and 8 weeks (Figure 2A and Table S2); moreover, the number of observed features did not significantly change between 1 and 8 weeks in either IG or CG. The overall composition of the maternal gut microbiome (β-diversity) was significantly different between IG and CG at 1 week (Figure 2B, Bray–Curtis distance, R^2^ = 0.04, *p* = 0.026), as determined using Bray–Curtis dissimilarity metrics. However, the separation between IG and CG was stronger at 8 weeks (Figure 2C, Bray–Curtis distance, R^2^ = 0.13, *p* = 0.001).

### 3.3. Microbiome Composition and Diversity in Breast Milk

The number of observed bacterial features in breast milk was not significantly different between IG and CG at 1 week, but it was significantly lower in IG relative to CG at 8 weeks (Figure 2D and Table S2, observed features 295 vs. 255, *p* = 0.032). However, the difference lost significance after adjusting for the 1 week baseline value (coefficient B = 37.5, 95%CI −42, 117, *p* = 0.3). The overall bacterial composition was not significantly different between IG and CG at 1 or 8 weeks, as determined using Bray–Curtis dissimilarity metrics (Figure 2E,F).

### 3.4. Microbiome Composition and Diversity in Infant Gut

Whilst no significant group differences were observed in the infant gut microbiome at 1 week, the IG infants presented a significantly higher evenness (Table S2 and Figure S4, Shannon index 1.94 vs. 2.27, *p* = 0.015) and a significantly higher number of observed bacterial features at 8 weeks (Figure 2G, observed features 73 vs. 113, *p* < 0.001); interestingly, the difference was still significant after adjusting for the 1 week baseline value (coefficient B = 40.8, 95%CI 15.7, 65.9, *p* = 0.002). The overall composition, as determined using Bray–Curtis dissimilarity metrics, was not significantly different between IG and CG at 1 (Figure 2H) or 8 weeks (Figure 2I).

### 3.5. Differences in Microbiome Composition in Maternal Gut, Breast Milk, and Infant Gut Samples between Groups

**Figure 3:**
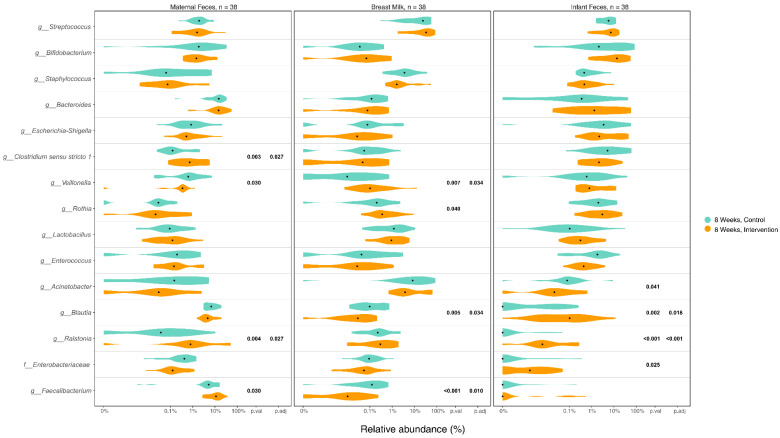
Relative abundance of the top 15 bacteria among maternal feces, breast milk, and infant feces samples. Notes: Relative abundance of the top 15 most abundant genera in maternal feces, breast milk, and infant feces samples were examined and the statistical differences between intervention and control groups were compared using the Wilcoxon rank-sum test and FDR adjusted *p*-value.

The top 15 most abundant bacteria in maternal gut, breast milk, and infant gut samples at 8 weeks after intervention were assessed, with group differences between IG and CG presented in Figure 3 and baseline group comparison presented in Figure S5 (Supplemental File). The relative abundance of some genera showed significant group differences at 8 weeks, with no group difference at baseline, such as lower *Veillonella* and higher *Faecalibacterium* in the IG maternal gut at 8 weeks. In breast milk, on the other hand, *Veillonella* was significantly higher in the IG at both baseline and 8 weeks. *Veillonella* was one of the common gut-associated obligate anaerobic genera shared between maternal gut, breast milk, and infant gut samples, whilst *Faecalibacterium* was commonly shared between maternal gut and breast milk samples. Two other gut-associated anaerobic genera, *Bifidobacterium* and *Blautia*, both had a higher relative abundance in the IG infant gut at 8 weeks, but this was only significant for *Blautia*. Moreover, although not significant, the relative abundance of *Bifidobacterium* in breast milk was lower in IG at baseline (Figures S5 and S6 in Supplemental File), but higher at 8 weeks after intervention (Figure 3). The relative abundance of two putative pathogens, *Enterococcus* and *Acinetobacter*, was also significantly lower in the IG at 8 weeks.

Notably, as a member of the *Proteobacteria* phylum, *Ralstonia* has commonly been identified in breast milk, but rarely in the gut [12,37]. However, the present study observed a significantly higher relative abundance of *Ralstonia* in IG maternal and infant gut at both baseline and 8 weeks.

### 3.6. Correlation between the Top 15 Microbial and Maternal Stress/Infant Weight

**Figure 4:**
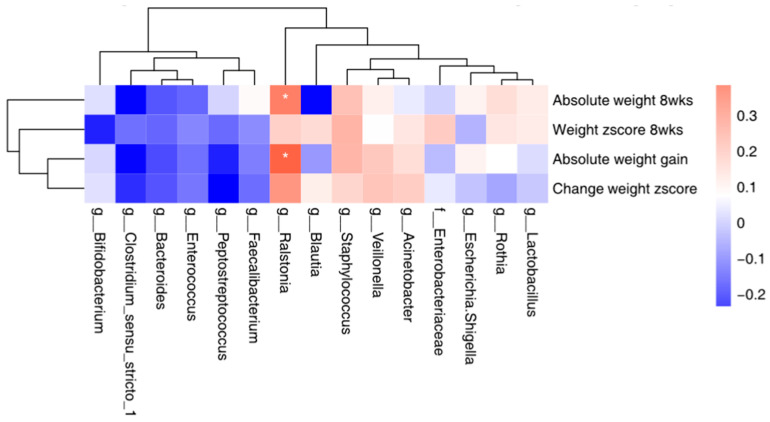
Top 15 bacteria in infant feces samples and its correlation with infant weight at 8 weeks and weight gain from 1 to 8 weeks. Notes: wks = weeks, weight z-score was calculated based on 21st intergrowth study preterm newborn database. * *p* < 0.05.

We examined whether the infant gut microbiome at 8 weeks was correlated with infant weight at 8 weeks and weight gain from 1 to 8 weeks, as well as with maternal stress at 8 weeks; the top 15 bacteria were included in the analyses. Higher abundance of *Ralstonia* in infant gut was significantly correlated with infant weight gain from 1 to 8 weeks (r = 0.38, *p* = 0.017) and higher absolute infant weight at 8 weeks (r = 0.33 *p* = 0.04). No significant correlation was identified between any of the 15 bacteria and maternal stress (Figure 4).

### 3.7. Unintended Effects

No unintended effects were reported by mothers included in this study.

## 4. Discussion

Using an experimental approach, our study suggests that a simple relaxation intervention for lactating mothers, aimed at mitigating maternal stress and enhancing infant weight gain, elicited changes in the microbiome composition and diversity across the maternal gut, breast milk, and infant gut. These findings are consistent with the proposed hypothesis, suggesting a potential role of the microbiome as a mediator of the observed effects of relaxation interventions through gut–brain and entero-mammary pathways. Key support for this finding comes from the differences in the microbial communities when comparing IG and CG of maternal gut, breast milk, and infant gut microbiomes.

Compared to baseline, the overall microbial community structures in the maternal gut showed a greater separation between the IG and CG at 8 weeks, suggesting that the microbiome community structure was altered after the intervention. Nevertheless, the number of observed taxa in the maternal gut did not significantly differ between IG and CG and did not change significantly from 1 to 8 weeks, underlining that the microbiome richness in the adult gut may be relatively stable. Comparatively, the observed features and evenness of the microbiome in the infant gut were significantly higher in the IG than the CG at 8 weeks. This may be regarded as beneficial, since studies have suggested that a higher infant gut α-diversity reflects a more mature, adult-like community [34,38]. It should be noted that, although non-significant, there were 47.4% CG mothers and 57.9% IG mothers who reported that they continued EBF at 8 weeks (10% difference between groups, *p* = 0.746), the remaining mothers were mostly breastfeeding, with breastfeeding accounting for more than 70% of feeds. It is possible that the higher α-diversity may partially reflect the introduction of formula or other fluids, since studies have shown a greater α-diversity in formula-fed infants than breastfed infants [39,40,41,42]. However, in our sample, IG infants presented a greater α-diversity, yet had a lower non-EBF rate compared to CG infants, conflicting with those published results; this suggested that non-EBF might not be the explanation for the significantly greater α-diversity in the IG infants’ gut at 8 weeks. Additionally, compared to the published data in term and preterm infants, the Shannon index at 8 weeks in the present study was similar to that of EBF infants in previous studies (around 2.0) [39,41,42], suggesting that the impact of other fluids on our results might be small. However, the interpretation of α-diversity is complex and can be affected by various factors, including geography [43] and the environment [44]. These findings, therefore, merit further investigation including metabolomics analysis applied to a larger sample.

As previously hypothesized, the changes in the infant gut microbiome could be related to the maternal gut microbiome though breast feeding. However, whilst the α-diversity was significantly higher in IG infant feces, it was significantly lower in IG breast milk. Apart from the potential impact of additional fluids other than breastmilk provided at 8 weeks on this finding, as discussed previously, some other hypotheses also merit consideration. On the one hand, a lower microbial diversity in breast milk could potentially be more consistent in its effects on infant health [45], since in a lower-diversity community, there might be stronger microbial competition. This competition could potentially inhibit the growth of harmful or pathogenic microorganisms, contributing to a healthier microbial balance in the infant’s gut [46]. On the other hand, whilst breastfeeding plays a key role in infant intestinal colonization [47], the infant gut microbiome does not share the composition and community structure seen in breast milk. Breast milk promotes a balanced microbiota development for the newborn, owing to its high content of unique oligosaccharides. These HMOs are ranked as the third most prevalent solid constituent in breast milk, after lactose and lipids, and play a pivotal role in fostering intestinal colonization within the infant gut [48,49]. Therefore, the content of HMOs in breast milk is an important factor in determining the microbiota diversity and composition in the infant gut. We did not measure the HMO content of breast milk in our trial. However, it is possible that HMOs were more abundant in the breast milk of IG mothers following the intervention and this merits further investigation, since previous evidence reported more abundant HMOs in mothers with good mental health compared to those who were distressed [50,51].

*Bifidobacterium* are predominant in the gut microbiota of infants and they are considered to be important for infant health and development [8,52,53]. *Bifidobacterium* in breast milk has been reported to activate immunoglobulin A (IgA)-producing plasma cells in the neonatal gut [54] and could control inflammation through mucosal host–microbe crosstalk [55]. The present study showed a lower relative abundance of *Bifidobacterium* in IG breast milk at baseline but a higher abundance after the relaxation intervention, suggesting the intervention may have increased the relative abundance of *Bifidobacterium* in IG breast milk. However, the increase in *Bifidobacterium* in the infant gut following the intervention was not as obvious as that in breast milk. This finding is in agreement with studies showing that *Bifidobacterium* colonizes the infant gut rapidly within the first few months [13,53]; although the CG infants showed a significantly lower baseline abundance than IG, it increased in both groups and no significant group difference was observed at 8 weeks. Overall, we suggest that the relaxation intervention contributed to an increase in *Bifidobacterium* in breast milk but had less of an impact on its colonization in the infant gut.

Modulation of the *Blautia* genera is worth noting. Although its role in infant gut homeostasis remains less known, studies in adult patients undergoing allogeneic hematopoietic stem cell transplantation for leukemia highlight a positive association of *Blautia* with a reduced rate of gut Graft versus Host Disease (GvHD) post-transplantation [56]. Future studies investigating potential crosstalk between beneficial bacteria that colonize the infant gut including *Bifidobacterium*, *Lactobacillus*, and *Blautia* genera are warranted.

The main published outcomes from our trial included a greater infant weight gain (mean difference in z-score = 0.51, 95%CI: 0.2, 0.9) and lower maternal stress (mean difference in Perceived Stress Scale = 2.7, 95%CI: 0.8, 4.5) in the IG at 8 weeks [26]. The correlation analysis in the present study further identified a significant association between a higher abundance of infant gut *Ralstonia* and a greater infant weight at 8 weeks. *Ralstonia* has commonly been observed in breast milk, whilst in the human gut, *Ralstonia* has mostly been reported as an opportunistic pathogen causing nosocomial infections in immunocompromised patients. In the present study, *Ralstonia* was rarely observed in the CG, yet a significantly higher relative abundance of *Ralstonia* was observed in IG maternal and infant gut samples at both baseline and 8 weeks, with a positive correlation between *Ralstonia* and infant weight gain and the absolute weight value at 8 weeks. Again, this finding merits further investigation, since *Ralstonia* can be a common contaminant of DNA extraction kits or PCR reagents, which may lead to its erroneous appearance in microbiota or metagenomic datasets [57]. However, it is less plausible that only IG gut samples would show contamination, since all samples were coded before being sent for analysis and the research assistants were unable to distinguish between the groups.

Our study has several limitations. Due to privacy concerns, the majority of samples were self-collected by mothers without supervision. Despite receiving clear instructions beforehand, this approach may have introduced contamination to the collected samples. Additionally, we did not collect samples from the mother or infant’s mouth, maternal areolar skin, or the vagina and all these potential sources could contribute additional bacteria to the infant gut microbiome. We also did not collect maternal dietary data, which could have influenced microbiome composition. However, all mothers were randomly assigned into relaxation or control groups with no difference in baseline characteristics between groups; moreover, mothers were following the traditional Chinese postpartum confinement practices with similar diet and lifestyle, thus reducing concerns of bias. Moreover, whilst all mothers were EBF at baseline, half of them did not continue EBF at 8 weeks. Although those mothers were still mostly breastfeeding and there was no significant difference regarding the EBF rate between IG and CG, it could have influenced microbiome diversity and structures in the infant gut microbiome. However, since the α-diversity was lower in CG infants’ gut samples and they had a higher non-EBF rate than IG infants, the greater α-diversity in the IG may not be explained by the non-EBF fluids. Due to the small sample size, we could not exclude non-EBF mother–infant pairs from our analysis and the results need to be confirmed in EBF mother–infant pairs in future studies with a larger sample size. Whilst the present analyses are somewhat limited by the relatively small sample size, the study sample was characterized by a high degree of homogeneity, as all mothers were primiparous Chinese women following vaginal delivery at 34–37 weeks, which increased the power to detect significant differences between IG and CG, reducing potential bias. Furthermore, it should be noted that although we randomly assigned mothers with no significant differences observed in baseline demographics, the baseline β-diversity was significantly different between IG and CG mothers’ gut samples, potentially due to the small sample size that individual outliers may have impacted on the overall results. Compared to baseline status, our results showed a stronger difference in gut microbiome diversity after the intervention, which may imply potential effects of the intervention. However, this should be treated with caution and needs to be further confirmed in larger trials.

## 5. Conclusions

This is the first study to test the hypothesis that the microbiome may serve as a signal between mother and infant during lactation, using an experimental approach. Through the implementation of the relaxation intervention to modify maternal psychological status, we could evaluate the causal relationship between maternal stress and maternal gut and breastmilk microbiome and its subsequent consequences for the infant gut microbiome. Consistent with our hypothesis, we found significant differences in microbiome composition and diversity between groups, together with observed differences in the enrichment of specific genera and correlations between biomarkers and clinical outcomes. These findings are best considered as hypothesis-generating and can inform the design of future studies. This could include larger trials in different populations, ideally with maternal dietary data, the collection of the additional biological samples mentioned above, and the application of metagenomic sequencing and metabolomics.

## Figures and Tables

**Table 1:** Baseline characteristics of the study participants.

Characteristics	Total N = 38	Control N = 19	Intervention N = 19
Mean (SD)			
Maternal age (yr)	31 (3)	30 (1.6)	31 (3.8)
Maternal education (yr)	16.0 (1)	15.5 (0.9)	16.4 (1.6)
Maternal BMI (kg/m^2^) **^a^**	23.3 (3)	22.6 (1.9)	24.0 (3.7)
**Maternal stress ^b^**	20.3 (8)	20.2 (7.1)	20.5 (8.9)
Infant weight (kg) ^c^	2.70 (0.3)	2.69 (0.3)	2.71 (0.4)
Infant length (cm) ^c^	47.7(2)	47.5(2)	48.0(2)
N (%)			
Gestational week			
34	1 (3)	0 (0)	1 (5.3)
35	4 (11)	2 (10.5)	2 (10.5)
36	17 (45)	8 (42.1)	9 (47.4)
37	16 (42)	9 (47.4)	7 (36.8)
N (%)			
Infant sex			
Male	16 (42)	7 (36.8)	9 (47.4)
Female	22 (58)	12 (63.2)	10 (52.6)
EBF			
1 week ^d^	38 (100)	19 (100)	19(100)
8 weeks ^e^	20 (52.6)	9 (47.4)	11 (57.9)
**Use of antibiotics ^f^**			
During hospital stay	3 (7.9)	2 (10.5)	1 (5.3)
During 1–8 weeks	0	0	0

Notes: SD = standard deviation. N = number. BMI = body mass index. EBF = exclusively breastfed, self-reported by mothers, with the definition provided on the questionnaire. ^a^ BMI calculated using maternal weight at 1 week home visit. ^b^ Maternal stress measured by using the Chinese version of the Perceived Stress Scale. ^c^ Weight and length were measured using standard anthropometry assessment at the 1 week home visit. ^d^ Infant could receive expressed breast milk or formula initially, but had to be EBF at 1 week enrolment. ^e^ Infants who were not exclusively breastfed at 8 weeks were still breastfed (with breast milk >70% of the feedings). ^f^ Three mothers took antibiotics during hospital stay, due to a vaginal incision. Two mothers in the control group took Cephalosporins and Amoxicillin, respectively; one mother in the intervention group took Cephalosporins. All mothers reported no medicine intake during the study period.

## Data Availability

All raw sequencing data associated with this study have been uploaded to the Sequence Read Archive (SRA) under citation accession PRJNA1000236. Data described in the manuscript, code book, and analytic code will be made available upon request due to.

## Supplementary Materials

The following supporting information can be downloaded at: https://www.mdpi.com/article/10.3390/nu16071074/s1, CONSORT checklist; Figure S1. Hypothesis of the potential mechanism of microbial transfer from mother to infant. Table S1. Comparisons of the demographic characteristics, maternal stress and infant weight gain between the selected and non-selected mother-infant pairs. Figure S2. Microbiome Analysis of Maternal Gut, Breast Milk, and Infant Gut samples Figure S3. Differences in microbiome community structures among maternal gut, breast milk, and infant gut. Table S2. Numbers of observed species (α-diversity) in breast milk, maternal stool, and infant stool microbiota and comparison between intervention and control group. Figure S4. Group differences in α-diversity based on Shannon index, ACE, and Chao1. Figure S5. Relative abundance of the top 15 bacteria in maternal feces, breast milk, and infant feces at baseline. Figure S6. Relative abundance of Bifidobacterium in maternal gut, breast milk, and infant gut by individuals.
