# Supplementary material for: A Stress Reduction Intervention for Lactating Mothers Alters Maternal Gut, Breast Milk, and Infant Gut Microbiomes: Data from a Randomized Controlled Trial
Source: Nutrients. 2024 Apr 6;16(7):1074. doi: 10.3390/nu16071074 (PMC11013067; doi:10.3390/nu16071074)

Supplementary Online Content

Figure S1. Hypothesis of the potential mechanism of microbial transfer from mother to infant.

Table S1. Comparisons of the demographic characteristics, maternal stress and infant weight gain between the selected and non-selected mother-infant pairs.

Figure S2. Microbiome Analysis of Maternal Gut, Breast Milk, and Infant Gut samples

Figure S3. Differences in microbiome community structures among maternal gut, breast milk, and infant gut

Table S2. Numbers of observed species (α-diversity) in breast milk, maternal stool, and infant stool microbiota and comparison between intervention and control group

Figure S4. Group differences in α-diversity based on Shannon index, ACE, and Chao1

Figure S5. Relative abundance of the top 15 bacteria in maternal feces, breast milk, and infant feces at baseline

Figure S6. Relative abundance of *Bifidobacterium* in maternal gut, breast milk, and infant gut by individuals

Figure S1. Hypothesis of the role played by the microbiome in the relationship between maternal stress and infant growth and behaviour.

Notes: EMT= entero-mammary trafficking; HMO=human milk oligosaccharides.

This figure describes how the microbiome could be involved in mother-infant interaction (signalling). During lactation, maternal gut microbiome could be affected by maternal stress via gut-brain axis, and further influence the breast milk microbiome by entero-mammary trafficking, leading to the shifts in infant gut microbiome through breastfeeding.

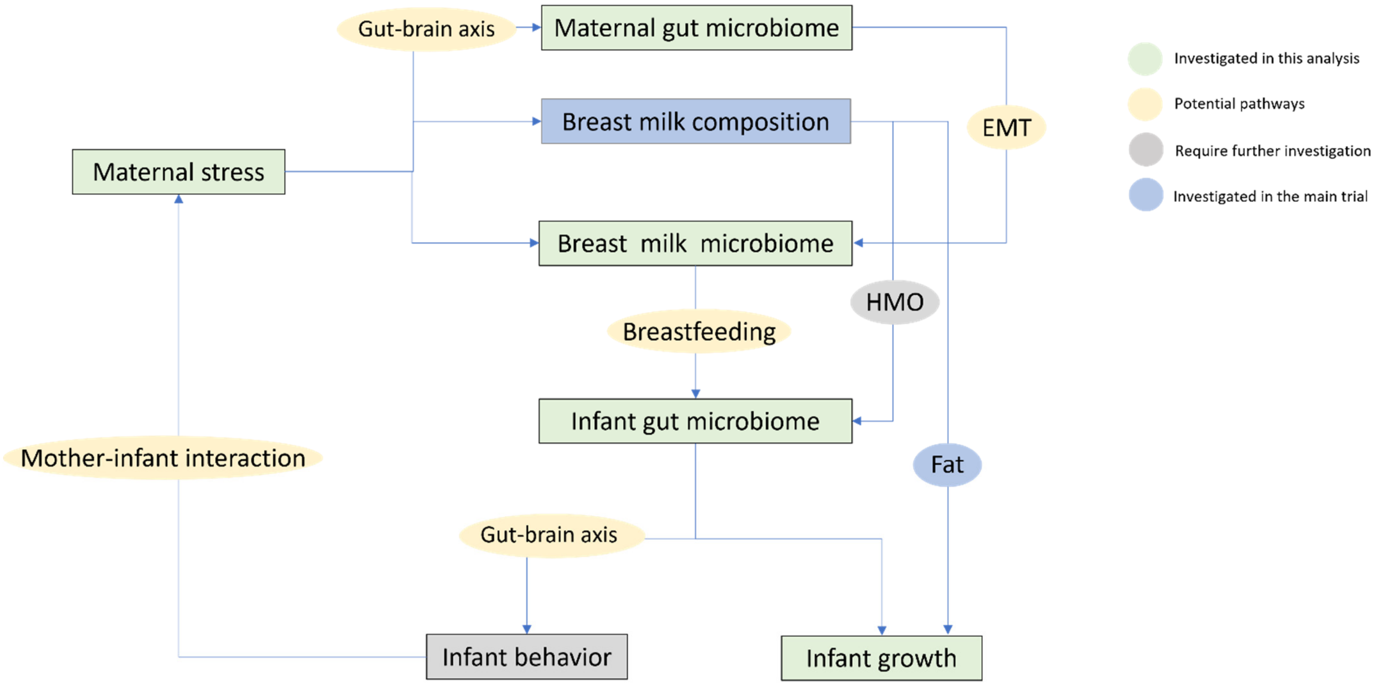

Table S1. Comparisons of the demographic characteristics, maternal stress, and infant weight gain between the selected and non-selected mother-infant pairs.

	Involved in analysis	Mean (SD)	t-test for Equality of Means					
				t	Sig. (2-tailed)	Mean Difference	95% CI	
							Lower	Upper
Gestational week	No (n=58)	36.00 (1.01)	Equal variances assumed	-1.372	0.173	-0.263	-0.64	0.12
	Yes (n=38)	36.26 (.76)	Equal variances not assumed	-1.454	0.149	-0.263	-0.62	0.10
Birth weight	No (n=58)	2.70 (.29)	Equal variances assumed	0.045	0.964	0.003	-0.12	0.12
	Yes (n=38)	2.70 (.30)	Equal variances not assumed	0.045	0.964	0.003	-0.12	0.13
Maternal age	No (n=58)	29.36 (3.61)	Equal variances assumed	-1.621	0.108	-1.138	-2.53	0.26
	Yes (n=38)	30.50 (2.95)	Equal variances not assumed	-1.691	0.094	-1.138	-2.48	0.20
Full time education	No (n=58)	15.35 (2.19)	Equal variances assumed	-0.309	0.758	-0.155	-1.15	0.84
	Yes (n=38)	15.50 (2.70)	Equal variances not assumed	-0.296	0.768	-0.155	-1.20	0.89
PSS at week 1	No (n=58)	20.02 (7.16)	Equal variances assumed	-0.191	0.849	-0.299	-3.40	2.80
	Yes (n=38)	20.32 (7.95)	Equal variances not assumed	-0.187	0.852	-0.299	-3.48	2.88
PSS at week 8	No (n=58)	18.93 (6.48)	Equal variances assumed	0.734	0.465	0.984	-1.68	3.64
	Yes (n=38)	17.95 (6.32)	Equal variances not assumed	0.738	0.462	0.984	-1.67	3.63
Change in PSS	No (n=58)	-1.09 (5.18)	Equal variances assumed	1.285	0.202	1.282	-0.70	3.26
	Yes (n=38)	-2.37 (4.09)	Equal variances not assumed	1.350	0.180	1.282	-0.61	3.17

Infant weight gain	No (n=58)	2.57 (.58)	Equal variances assumed	0.379	0.706	0.044	-0.19	0.27
	Yes (n=38)	2.52 (0.51)	Equal variances not assumed	0.390	0.697	0.044	-0.18	0.27
Chi-Square Test								
Infant sex	Involved male	16 (42%)	Fisher's Exact Test (2-sided)					
	Non-involved male	36 (62%)	0.063					

Notes: PSS=perceived stress scale; CI=confidence interval; SD=standard deviation.

Figure S2. Microbiome Analysis of Maternal Gut, Breast Milk, and Infant Gut samples

Notes:The distribution of read counts in maternal gut after standard sequencing quality control between control and intervention group at 1 week and 8week as shown in A; the distribution in breast milk as shown in B; the distribution in infant gut as shown in C; rarefaction curve as shown in D shown subsampled feature counts for each sample among maternal gut, breast milk, and infant gut, using a sampling depth of number 80000. CG=control group, IG=intervention group, BM=breast milk, IS=infant gut, MS=maternal gut.

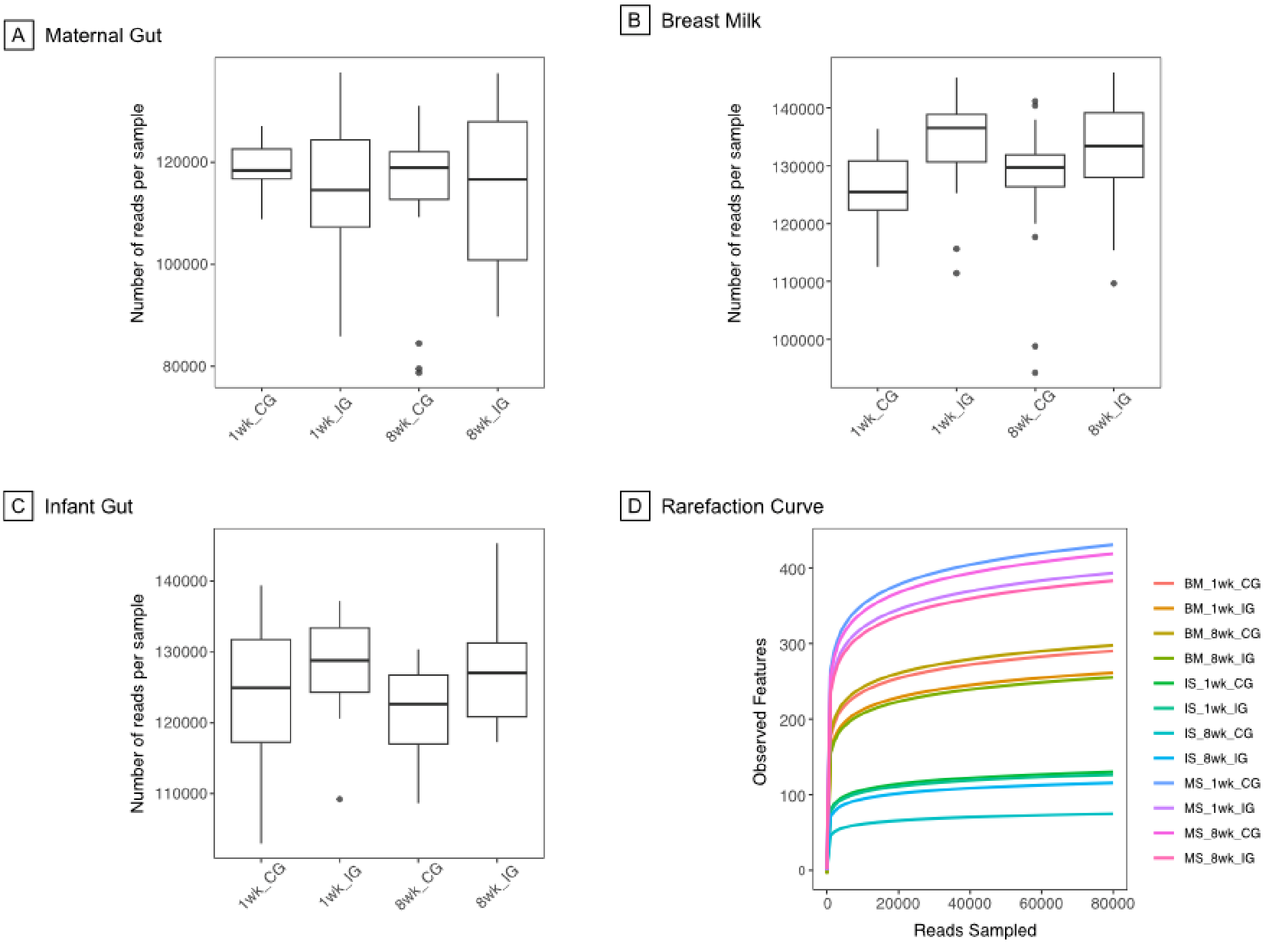

Figure S3. Differences in microbiome community structures among maternal gut, breast milk, and infant gut.

Notes: The microbiome community difference among maternal gut, breast milk and infant gut were measured using Bray-Curtis distance matrix, and presented using principal coordinates analysis plot (PCoA). Statistical difference was assessed by using analysis of similarities (ANOSIM).

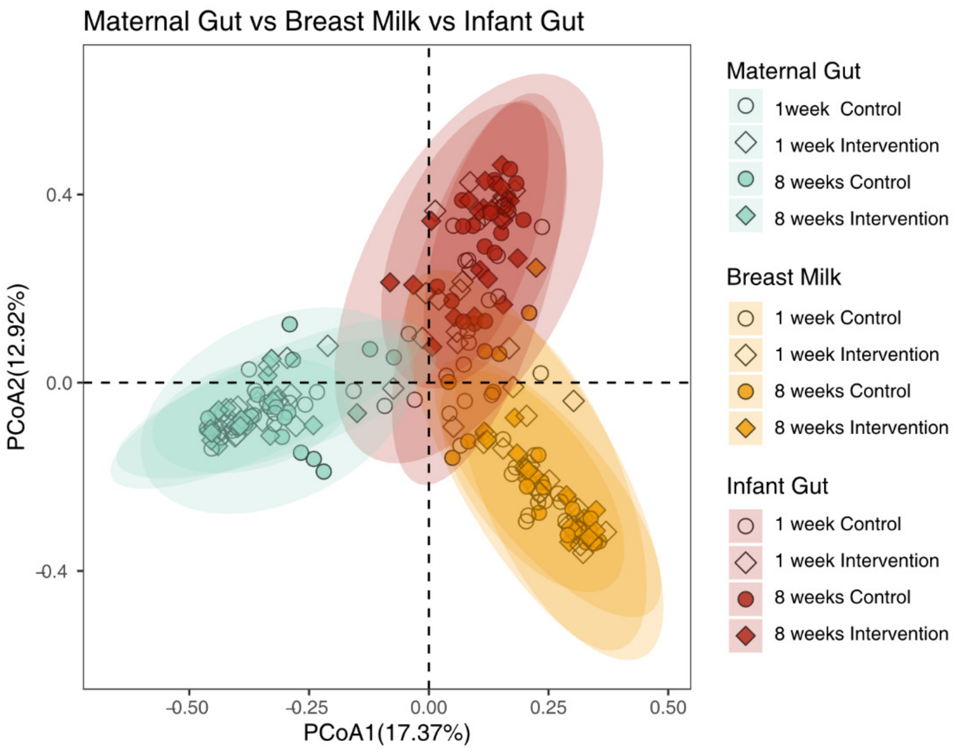

Table S2. α-diversity in breast milk, maternal stool, and infant stool microbiota and comparison between intervention and control group.

				Observed Features	Shannon	ACE	Chao 1
**1-week**	Maternal gut	Median (IQR)	CG	374(124)	4.24(1.1)	379(125)	381(123)
			IG	366(140)	3.93(0.5)	370(139)	372(139)
		Sig. (2-sided)		0.74	0.45	0.69	0.71
	Breast milk	Median (IQR)	CG	286(152)	2.62(1.0)	295(158)	298(159)
			IG	237(89)	2.77(1.0)	247(247)	250(84)
		Sig. (2-sided)		0.56	0.75	0.56	0.51
	Infant gut	Median (IQR)	CG	68(101)	1.90(0.8)	69(105)	69.5(105)
			IG	104(67)	2.28(0.8)	105(67)	104(68)
		Sig. (2-sided)		0.21	0.12	0.2	0.2
**8-weeks**	Maternal gut	Median (IQR)	CG	399(97)	4.14(0.7)	405(98)	406(98)
			IG	368(86)	4.08(0.6)	371(86)	370(86)
		Sig. (2-sided)		0.67	0.3	0.71	0.71
	Breast milk	Median (IQR)	CG	296(110)	3.03(0.4)	304(111)	302(113)
			IG	221(56)	2.87(0.5)	229(57)	228(59)
		Sig. (2-sided)		**0.032**	0.16	**0.037**	**0.027**
	Infant gut	Median (IQR)	CG	62(24)	1.94(0.7)	62(24)	62(24)
			IG	102(69)	2.27(0.3)	106(73)	106(75)
		Sig. (2-sided)		**<0.001**	**0.015**	**<0.001**	**<0.001**

Notes: IG=intervention group; CG=control group. Maternal and infant gut microbiome were examined using their feces sample. Significance tested using Wilcoxon Rank-Sum Test.

Figure S4. Group differences in α-diversity based on Shannon index, ACE, and Chao1.

Notes: Alpha diversity was measure using the Shannon index, ACE and chao1 in the maternal gut at 1 and 8 weeks, as shown in A; alpha diversity in breast milk is illustrated in B; alpha diversity in infant gut is depicted in C. Group differences in alpha diversity were assessed using Wilcoxon rank-sum test.

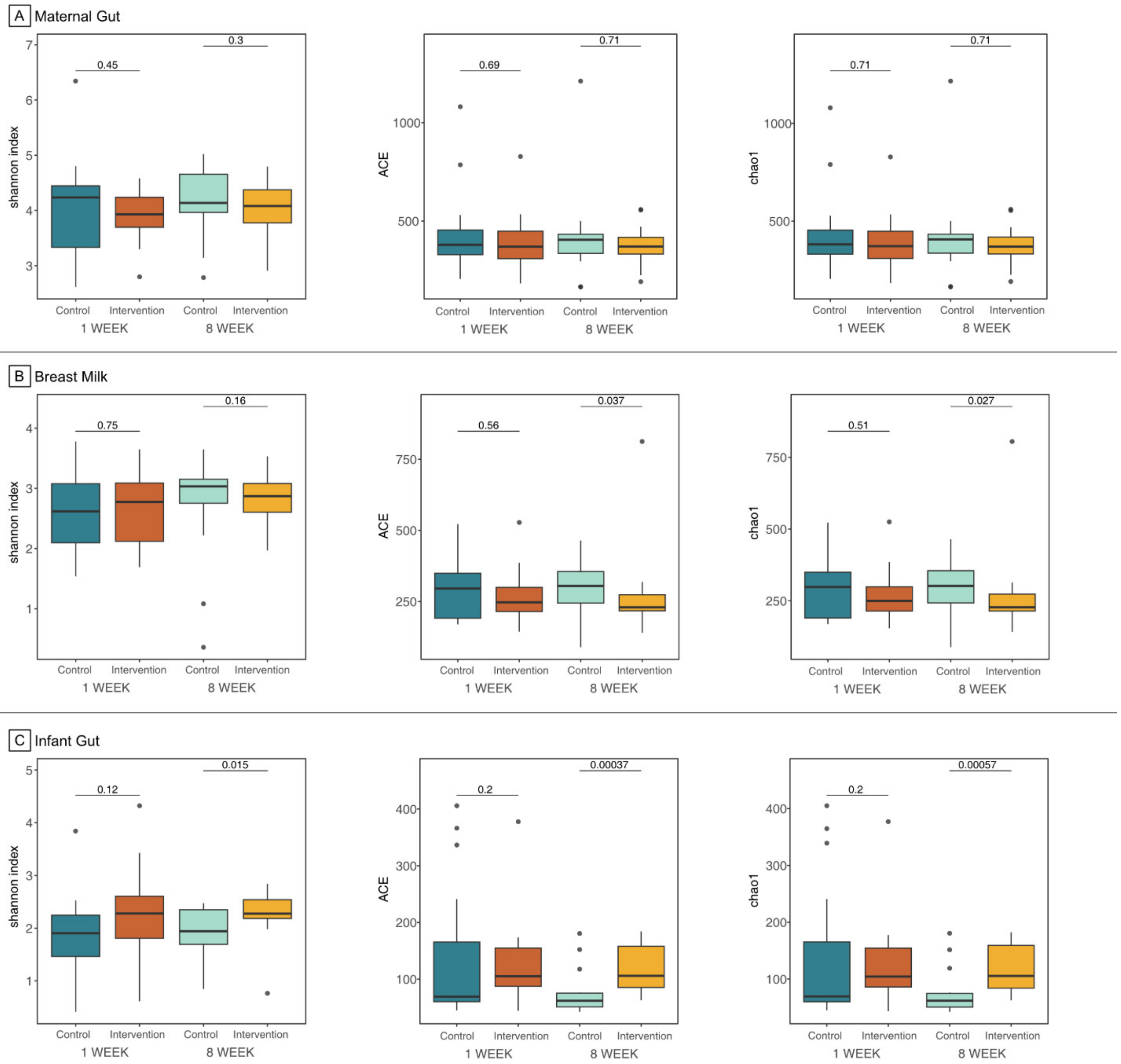

Figure S5. Relative Abundance of The Top 15 Bacteria in Maternal Feces, Breast Milk, and Infant Feces at Baseline.

Notes: The relative abundance of the top 15 most abundant genera in maternal feces, breast milk, and infant feces at baseline was examined. Statical difference between the intervention and control groups were compared using the Wilcoxon rank-sum test, and adjusted using the FDR method. CG=control group; IG=intervention group.

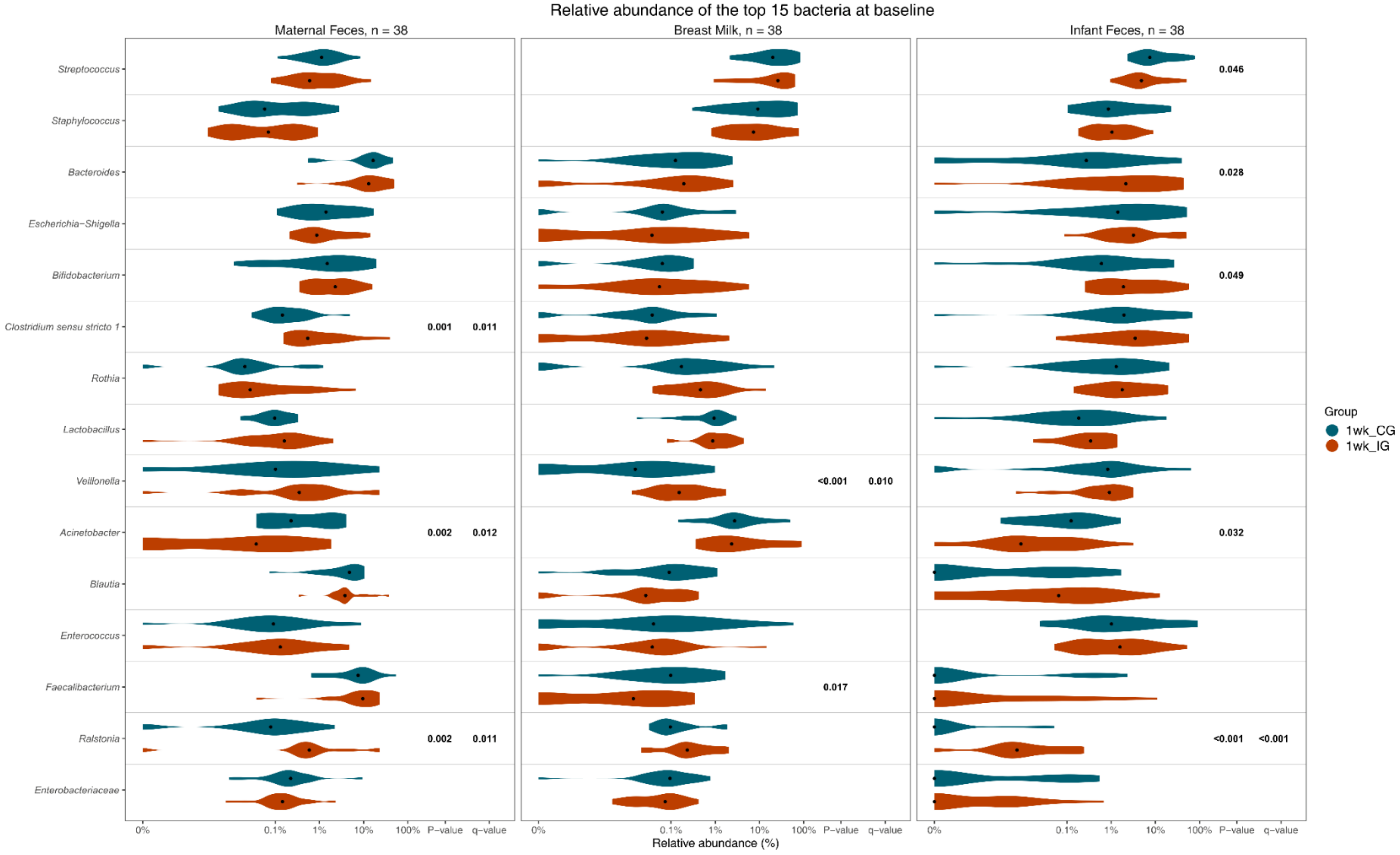

Figure S6. Relative Abundance of Bifidobacterium in Maternal Gut, Breast Milk, and Infant Gut by Individuals.

Notes: The figure displays the relative abundance of *Bifidobacterium* for each individual (with unique study ID) in the maternal gut, breast milk and infant gut at 1 and 8 weeks, comparing the control and intervention groups. MS=maternal gut, BM=breast milk, IS=infant gut.

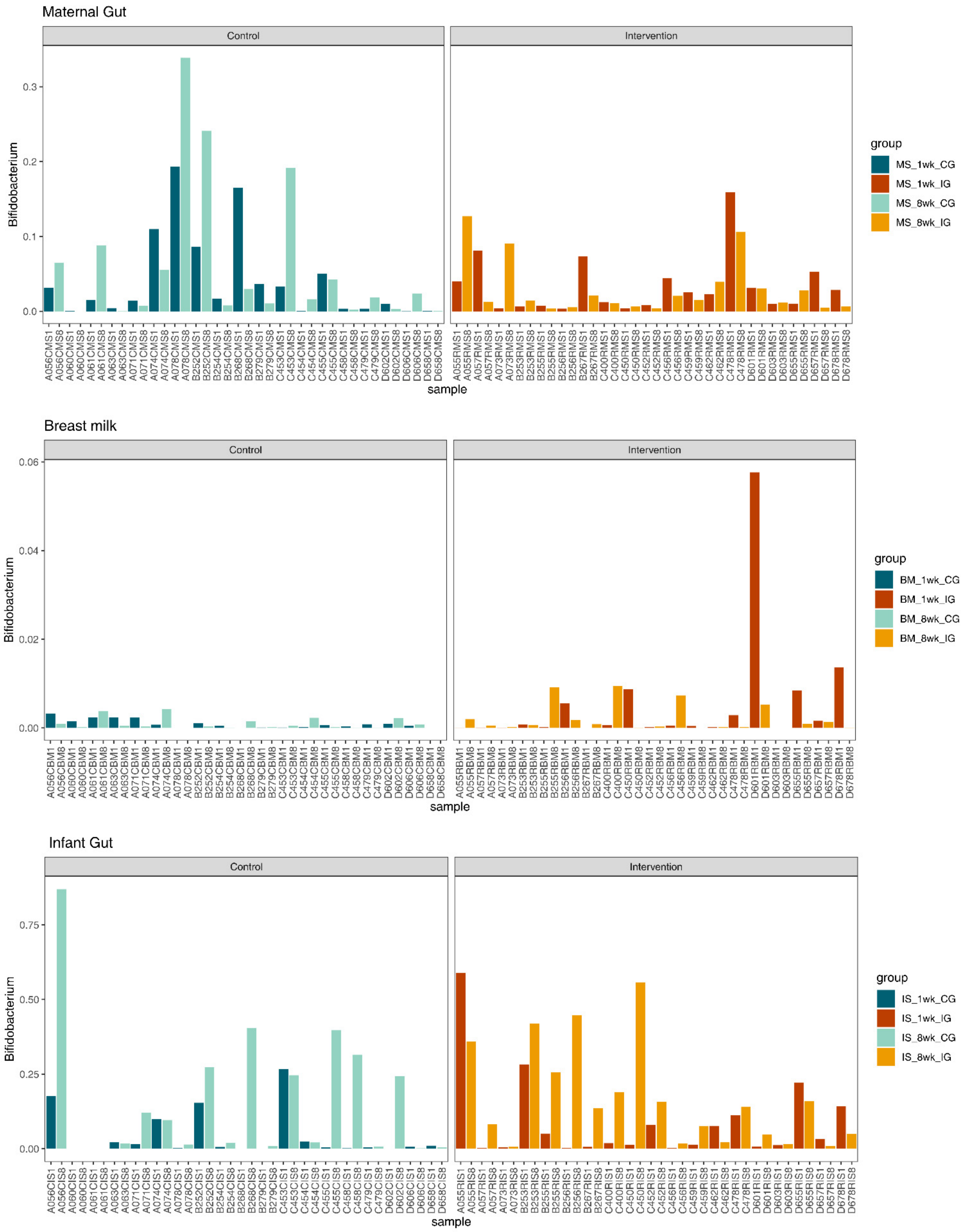

Supplement: Supplementary file 1 [file nutrients-16-01074-s001.zip › nutrients-2926797-supplementary.pdf]
